# Reproduction and Growth in a Murine Model of Early Life-Onset Inflammatory Bowel Disease

**DOI:** 10.1371/journal.pone.0152764

**Published:** 2016-04-05

**Authors:** Eniko Nagy, Ramona M. Rodriguiz, William C. Wetsel, Nancie J. MacIver, Laura P. Hale

**Affiliations:** 1 Department of Pathology, Duke University Medical Center, Durham, North Carolina, United States of America; 2 Department of Psychiatry and Behavioral Sciences, Duke University Medical Center, Durham, North Carolina, United States of America; 3 Department of Pediatrics, Duke University Medical Center, Durham, North Carolina, United States of America; 4 Mouse Behavioral and Neuroendocrine Analysis Core Facility, Duke University Medical Center, Durham, North Carolina, United States of America; Max Rubner-Institut, GERMANY

## Abstract

Studies in transgenic murine models have provided insight into the complexity underlying inflammatory bowel disease (IBD), a disease hypothesized to result from an injurious immune response against intestinal microbiota. We recently developed a mouse model of IBD that phenotypically and histologically resembles human childhood-onset ulcerative colitis (UC), using mice that are genetically modified to be deficient in the cytokines TNF and IL-10 (“T/I” mice). Here we report the effects of early life onset of colon inflammation on growth and reproductive performance of T/I mice. T/I dams with colitis often failed to get pregnant or had small litters with pups that failed to thrive. Production was optimized by breeding double homozygous mutant T/I males to females homozygous mutant for TNF deficiency and heterozygous for deficiency of IL-10 (“T/I-het” dams) that were not susceptible to spontaneous colon inflammation. When born to healthy (T/I-het) dams, T/I pups initially gained weight similarly to wild type (WT) pups and to their non-colitis-susceptible T/I-het littermates. However, their growth curves diverged between 8 and 13 weeks, when most T/I mice had developed moderate to severe colitis. The observed growth failure in T/I mice occurred despite a significant increase in their food consumption and in the absence of protein loss in the stool. This was not due to TNF-induced anorexia or altered food consumption due to elevated leptin levels. Metabolic studies demonstrated increased consumption of oxygen and water and increased production of heat and CO_2_ in T/I mice compared to their T/I-het littermates, without differences in motor activity. Based on the clinical similarities of this early life onset model of IBD in T/I mice to human IBD, these results suggest that mechanisms previously hypothesized to explain growth failure in children with IBD require re-evaluation. The T/I mouse model may be useful for further investigation of such mechanisms and for development of therapies to prevent reproductive complications and/or growth failure in humans with IBD.

## Introduction

Inflammatory bowel disease (IBD) is a chronic inflammatory disorder that is hypothesized to result from an inappropriate immune response against intestinal microbiota that occurs in a genetically predisposed host. Two forms of IBD are generally recognized in humans. The first, Crohn’s disease (CD), can affect any part of the gastrointestinal tract from mouth to anus and is characterized by areas where normal tissue is interspersed with affected tissue (skip lesions), transmural inflammation, and frequently by granulomas. The second form of IBD, ulcerative colitis (UC), is limited to the colon. Inflammation in UC always involves the rectum (proctitis) and can extend proximally in a continuous fashion to also involve more proximal colonic segments or the entire colon (pancolitis). Inflammation in UC is typically limited to the mucosa and crypt abcesses are common.

The genetic causes of IBD are only partly understood. Studies in transgenic mouse models and genome-wide genetic linkage and association studies in humans have provided insight into the genetic complexity underlying these inflammatory conditions. Many of the IBD susceptibility genes that have been identified are associated with both CD and UC [1]. This suggests common mechanisms in the pathogenesis of both diseases, particularly mechanisms that involve dysregulation or disruption of the innate or adaptive arms of the immune system and responses to microbes. Mutations in cytokine genes and/or their receptors have been specifically implicated in early onset (childhood) IBD. These include mutations in the TNF [1] and the IL-10 signaling pathways [1–4]. For example, mutations in the coding region of the IL-10 receptor that render patient cells non-responsive to IL-10 have been demonstrated in infants with very early onset of severe and treatment-resistant CD [3]. Specific polymorphisms in the TNF promoter that affect binding of transcription factors also predispose to IBD development in humans [5].

Cohort studies show that in contrast to adults, most children with UC present with or develop pancolitis rather than isolated inflammation of the rectum [6–8]. Weight loss or failure to gain weight is a presenting symptom in as many as 65% of children with UC [6]. This has been attributed to decreased oral intake due to anorexia (perhaps due to systemic circulation of pro-inflammatory cytokines such as TNF), early satiety, nausea, or pain [6]. Nutritional impairment and/or elevated levels of cytokines can also lead to a missed or delayed pubertal growth spurt and reduced final height, abnormal bone mineralization, and maintenance of pre-pubertal sex hormone levels [6]. Understanding and developing treatments that can counter the specific mechanisms that underlie these pathologies is critical, since affected children will require IBD treatment for decades.

Most of the current single gene knockout murine models of IBD result in a CD-like phenotype. We recently developed a mouse model of IBD using mice that are deficient in the cytokines TNF and IL-10 (“T/I “mice) [9]. We previously showed that T/I mice spontaneously developed severe colitis soon after weaning, without the need for exogenous triggers. Colitis in T/I mice had clinical and histologic features similar to human UC, including pan-colitis limited to the mucosa of the colon, elevated serum Th17-type cytokines, and a markedly increased risk of developing inflammation-associated colon cancer. Development of spontaneous colitis in these mice was microbiota-dependent and could be prevented or delayed by antibiotic treatment [9]. The purpose of the current studies was to define the effect of early life onset of colon inflammation on growth and reproduction of T/I mice.

## Materials and Methods

### Animal Studies

Mice with various levels of genetic deficiency of TNF and IL-10 were generated by cross-breeding pairs of mice deficient in IL-10 (strain name = *B6*.*129P2-Il10*^*tm1Cgn*^*/J*; stock # 002251; Jackson Laboratories, Bar Harbor, ME) with mice deficient in TNF (strain name = *B6*.*129S6-Tnf* ^*tm1Gkl*^*/J*; stock # 005540; Jackson Laboratories). Both of these strains were on the C57BL/6 background. For some studies, C57BL/6 mice (strain name = C57BL/6J; stock #000664; Jackson Laboratories) were used as wild type (WT) controls. The initial breeding scheme used *Tnf* ^*-/-*^; *Il10*^*-/-*^ double knockout (T/I) male and female breeders that were IBD-susceptible, with complete deficiency of TNF production in all cell types. This was later changed to breeding male T/I mice with female mice with heterozygous deficiency of TNF production and complete deficiency of IL-10 production (*Tnf* ^+/-^; *Il10*^-/-^, “T-het/I”). The final breeding scheme used male T/I mice and *Tnf* ^-/-^; *Il10* ^+/-^ (“T/I-het”) female mice. This latter breeding scheme generated 50% T/I-het and 50% T/I mice as littermates that would be exposed to the same maternal microbiota at birth. When possible, co-housed same-sex littermates of differing genotypes were used when age-matched comparisons were made. However, each genotype group was composed of multiple litters and the data obtained were not specifically matched to littermates.

Mice were housed in polycarbonate micro-isolator cages in individually ventilated racks under specific pathogen-free barrier conditions, with access to food (PicoLab Mouse Diet 20/5058, LabDiet, St. Louis, MO, USA) and water *ad libitum*. Sentinel mice exposed repetitively to dirty bedding from the mice used in this study were negative for parasites by microscopic examination, negative for *Citrobacter rodentium* by fecal culture, negative for infection with *Helicobacter* species by PCR of feces and negative by serology for a panel of 22 murine protozoal, bacterial and viral pathogens, including murine parvovirus, murine hepatitis virus, and murine norovirus (S1 Table).

Cohorts of mice were euthanized for tissue collection at predetermined time points up to 28 weeks of age or when they reached the humane endpoints of rectal prolapse, loss of >15% body weight, or signs of pain and distress including poor grooming, decreased activity, and hunched posture.

For studies of growth and food consumption, mouse weight and the food consumed per cage was determined 3 times per week from weaning to 28 wks of age. The amount of food consumed was normalized to g food/g body weight and was compared separately for males and females of WT, T/I-het, and T/I genotypes.

For studies of growth under conditions that inhibit or delay development of colitis, antibiotic therapy was initiated in T/I and T/I-het mice at the time of weaning, using commercially available rodent chow that contained 3 mg amoxicillin, 0.5 mg clarithromycin, 1 mg metronidazole, and 20 μg omeprazole per 5 g wafer (Bio-Serv, Frenchtown, NJ). Control groups were given identical wafers that lacked antibiotics.

### Tissue Analysis

After euthanasia, the colon was divided into 5 parts for histologic examination: cecum, proximal, mid, distal, and terminal colon/rectum, each identified using permanent tissue dyes (Bradley Products, Bloomington, MN). Tissues were fixed in Carnoy’s solution for 2–4 hrs or in 10% neutral buffered formalin for 18 hrs, then processed into paraffin blocks. The severity of inflammation seen in hematoxylin and eosin-stained sections was scored by a board-certified pathologist blinded to genotype or experimental group. Histologic scores were calculated as described [10,11], using a scale from 0–75 that takes into account mucosal changes in the 5 different bowel segments, including hyperplasia and ulceration, degree of inflammation, and % of each bowel segment affected by these changes. Using this scale, a score <12 indicates the absence of colitis, 13–24 indicates mild colitis, and ≥ 25 indicates moderate to severe colitis.

### Cytokine Studies

Measurement of serum cytokines was performed in the Duke Regional Biocontainment Laboratory Immunology Unit (Durham, NC) under the direction of Dr. Gregory D. Sempowski, using Luminex bead-based multiplex immunoassays. For data analysis, all values falling below the lower limits of quantitation (LLOQ) were replaced with the midpoint between zero and the LLOQ. Serum leptin levels were measured using the Leptin Mouse Quantikine ELISA kit (R&D Systems, Minneapolis, MN) as per the manufacturer’s instructions.

### Studies of Nutrient Utilization

Stool was collected by placing each mouse into an individual collection container under direct observation until 1–2 pellets were produced. Stool pellets were weighed, then stored at -20°C until used. Extracts were made by vortexing stool with phosphate-buffered saline containing proteinase inhibitors (Halt Protease Inhibitor Cocktail + EDTA, Thermo Scientific, Rockford, IL), at a concentration of 100 mg stool per ml buffer. The protein content of stool extracts was measured using the Pierce BCA Protein Assay Kit (Thermo Scientific) according to the manufacturer’s instructions. The water content of stool was measured indirectly by computing the weight difference between stool when fresh versus after drying for 72 hrs at 37°C.

### *In vivo* Metabolic Testing

Metabolic testing was performed by the Mouse Behavioral and Neuroendocrine Analysis Core Facility at Duke University (Durham, NC), using the Columbus Lab Animal Monitoring System (CLAMS) (Columbus Instruments; Columbus, OH). The CLAMS metabolic chamber incorporates an open circuit calorimeter combined with 24-hour, automated, simultaneous, non-invasive collection of multiple physiological and behavioral parameters, including motor activity, food and water consumption, metabolic performance, and temperature. Mice were maintained on a 12 hr:12 hr light/dark cycle and allowed to consume their normal diet and water *ad libitum*. To minimize variation due to sex or estrous cycle, all mice used for this portion of the study were male. 8–9 wk old T/I mice and their T/I-het littermates (n = 8 per genotype) were allowed to acclimate to individual housing in the CLAMS unit for 4 days prior to recording metabolic data for 72 hrs. These habituation data are displayed in S1 Fig. Data were analyzed using the CLAMS Examination Tool (CLAX), a statistical analysis program designed for use on data acquired from CLAMS. Body temperatures were obtained once per week, from weaning until the CLAMS studies were performed, using a clinical-grade non-contact infrared thermometer aimed at the internal surface of the ear pinna. After completion of the CLAMS studies, non-fasting measurements of blood sugar were made, using the Freestyle Lite blood glucose monitoring system (Abbot Diabetes Care, Almeda, CA).

### *In vitro* Metabolic Testing

CD4+ T cells were isolated from spleen and mesenteric lymph nodes in T/I-het and T/I mice by negative selection (StemCell Technologies, Vancouver, BC, Canada). T cell oxygen consumption rates (OCR) and extracellular acidification rates (ECAR) were measured with an extracellular flux analyzer (Seahorse Bioscience, North Billerica, MA). Spare respiratory capacity (SRC) was calculated by subtracting basal respiration rate from maximal respiration rate, using the XF Cell Mito Stress Test report generator.

### Statistical Analysis

The data are presented as means and standard error of the mean. Statistical comparisons of serum cytokines, chemokines, and leptin and stool protein in T/I-het vs. T/I mice were performed using Student’s t-test. Statistical comparisons of food consumption, breeding success, and body weights for the genotypes were performed using ANOVA and a *post-hoc* test for multiple comparisons using GraphPad Prism software, version 5.03 (GraphPad, La Jolla, CA). A Dunnett’s *post-hoc* test was used when each group was compared against the WT control. A Tukey’s *post-hoc* test was used when the experimental groups were compared against each other. The Kruskal-Wallis non-parametric test with correction for multiple comparisons was used for statistical analysis of histologic scores. CLAMS data were normalized to mouse weight and the data obtained each hour were averaged over the 72 hour test period for each mouse. Means for each genotype data were then compared using an independent samples (Student’s) t-test, using the CLAX software package. For all studies, a p value ≤ 0.05 was considered to represent a significant difference between groups.

### Ethics Statement

All animal studies were approved by the Institutional Animal Care and Use Committee of Duke University, an institution accredited by the Association for Assessment and Accreditation of Laboratory Animal Care (AAALAC), International. The applicable protocol numbers were A151-09-05, A093-12-04, A043-15-02, and A179-14-07. Although colitis has the potential to produce pain and distress, analgesics were not employed in these studies, since non-steroidal anti-inflammatory agents (NSAIDs) can exacerbate colitis and opioids affect intestinal motility and function. Suffering was minimized by providing euthanasia for mice that met humane endpoints, as specified in these protocols.

## Results

### Effect of IBD Susceptibility on Reproduction

**Fig 1:**
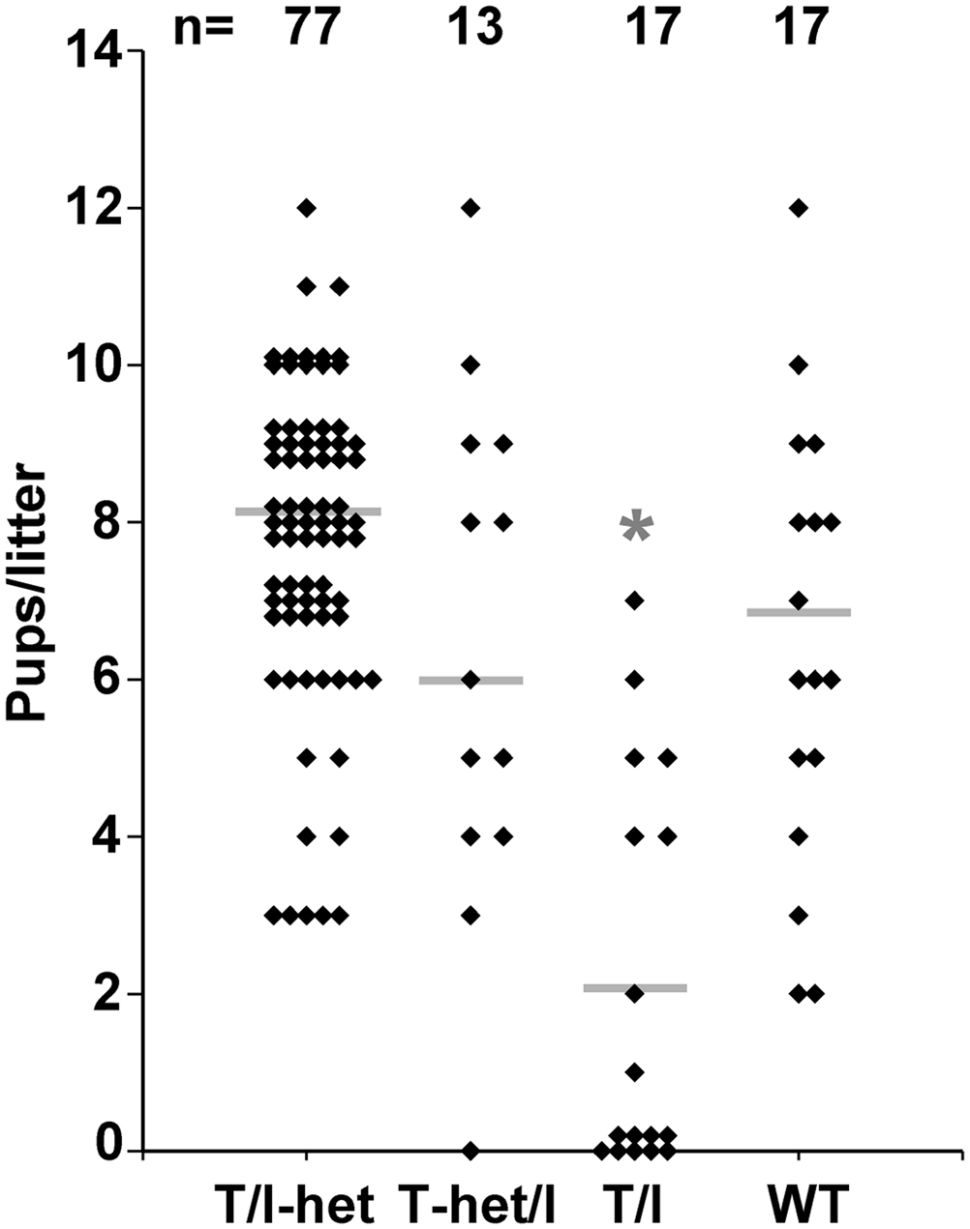
Reproductive success of dams with varying levels of TNF and IL-10 deficiency. Each point represents results of a single mating for the indicated dam genotypes. The mean number of pups/litter is indicated with a line. * indicates significant difference from WT (p < 0.0001; ANOVA with Dunnett’s post-test).

**Fig 2:**
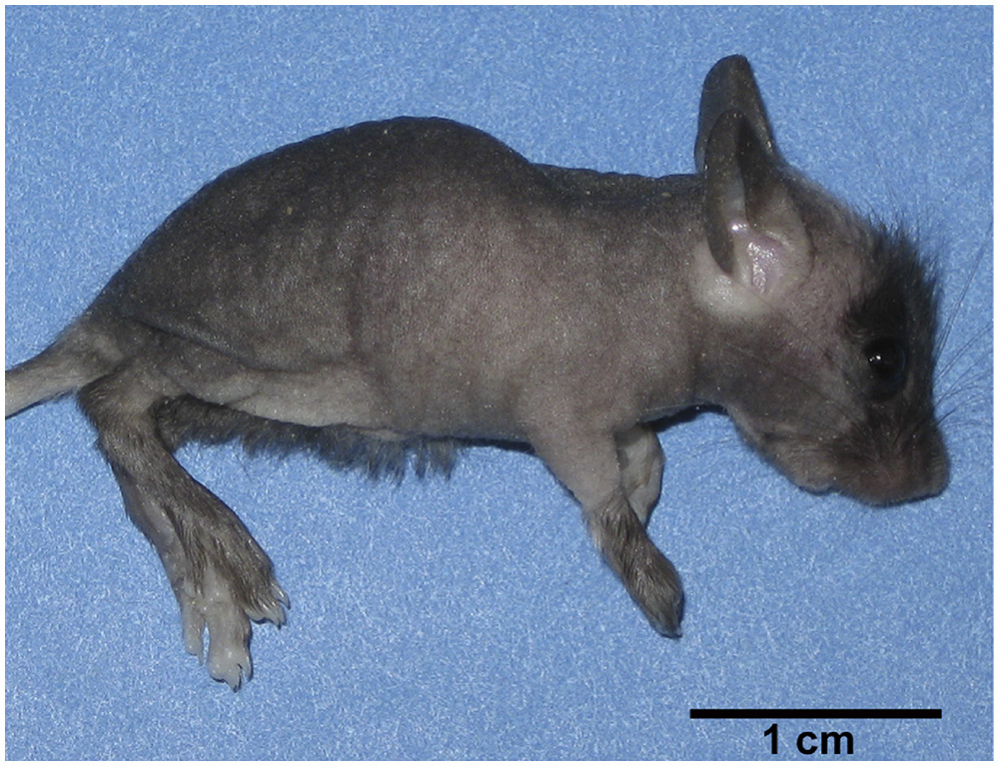
Growth retardation and alopecia in a T/I pup, 24 days old, produced and nursed by a T/I dam. Hair is retained on the face and sparsely on the lower abdomen, with near total hair loss in other body regions.

During initial studies, double knockout T/I males were bred with double knockout T/I females in an attempt to maximize the number of T/I pups produced. However, it rapidly became clear that T/I females often failed to get pregnant despite being co-housed with T/I males for prolonged periods of time. The total pups produced from 17 matings was 34, an average of 2 pups/litter (range 0–7; p < 0.0001 versus WT; Fig 1). The pups that were produced by T/I dams were visibly smaller than WT pups and typically failed to thrive. They also commonly developed alopecia (6 of 8 litters, 75%; Fig 2), a phenotype that we and others have previously demonstrated to be associated with iron deficiency [12,13]. Although histology was not routinely performed on failed or retired breeders, we previously showed that 78% of T/I mice (n = 23) examined during the typical breeder ages of 8–20 weeks had moderate to severe colitis [9].

In an attempt to enhance breeding success, double knockout T/I males were bred with T-het/I females, a scheme that generated 50% T-het/I and 50% T/I mice/litter. T-het/I mice also develop colitis of similar severity as seen in T/I mice, but we had previously observed that only 42% of T-het/I mice (n = 33) developed moderate to severe colitis between ages of 8–20 weeks [9]. This suggested that T-het/I dams were more likely to be in good health at the time of breeding compared with T/I females. T-het/I dams became pregnant and produced similar numbers of pups (6 ± 3) as did WT mice (7 ± 3; Fig 1). However, pup development of alopecia or failure to thrive still occurred regularly with T-het/I dams (8 of 11 litters, 73%).

**Fig 3:**
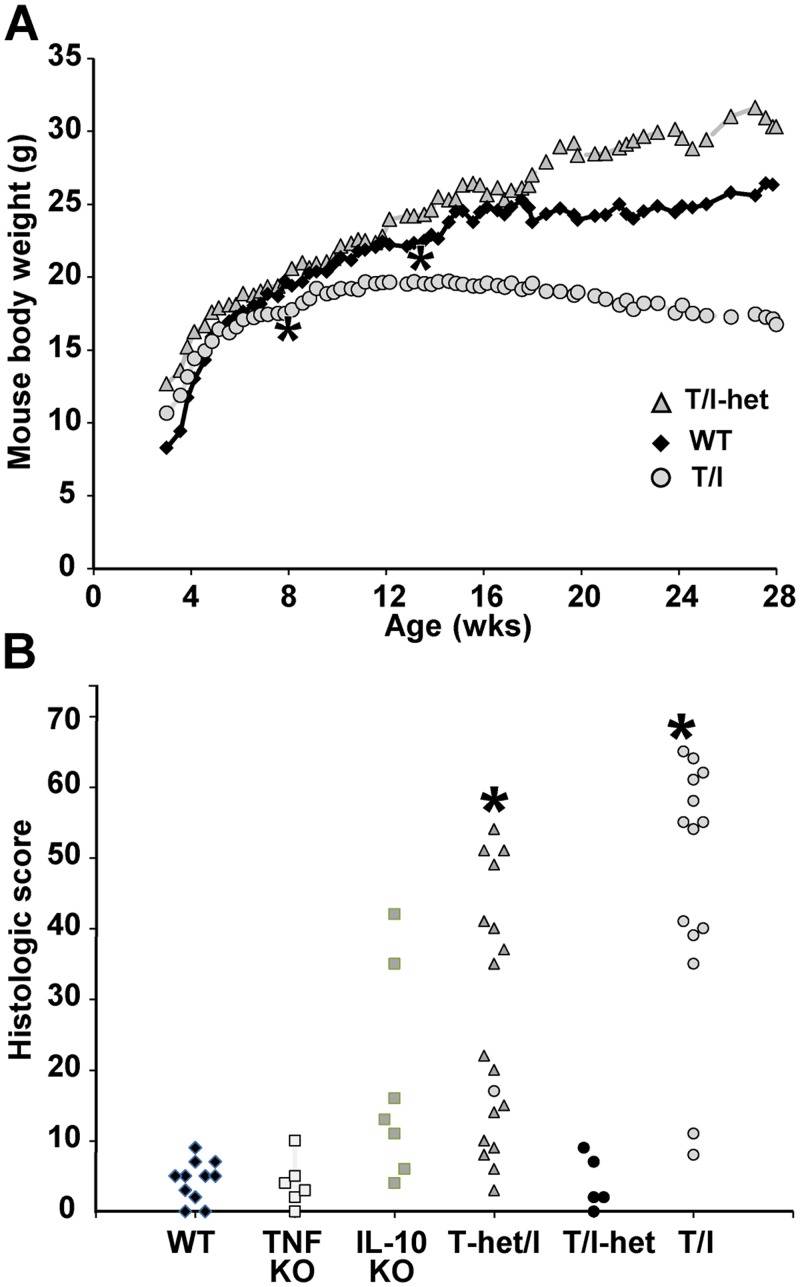
Effect of TNF and IL-10 deficiency on growth. A. Mean body weight is shown for female mice of the indicated genotypes from weaning until 28 weeks of age (n = 5 for T/I-het and T/I mice; n = 3–5 for WT, with 65 measurements per mouse). Although weights for all 3 genotypes were statistically similar at weaning, the weights of the T/I mice became significantly lower than their T/I-het littermates beginning at 8 wks and was lower than the WT mice beginning at 13 wks and they remained significantly lower for the remainder of the study (p < 0.05, 2 way ANOVA with Dunnett’s post-test). Similar trends were seen for male mice. SEMs averaged 0.8 g across all days and groups; error bars are omitted for clarity. B. Colitis histologic scores are shown for cohorts of 9–12 wk old mice. Each point represents a single mouse studied: WT (n = 11), *Tnf* ^-/-^ (n = 6), *Il10* ^-/-^ (n = 7), T-het/I (n = 17), T/I-het (n = 5), and T/I (n = 14). A score of ≤12 indicates absence of colitis, scores ≥ 25 indicate moderate to severe colitis, and the maximum possible score is 75. * indicates a significant difference from WT; p ≤ 0.0001 for both T-het/I and T/I (Kruskal-Wallis non-parametric test with correction for multiple comparisons).

In an attempt to further enhance breeding success, T/I-het females were generated and bred to T/I males, a scheme which generated 50% T/I-het and 50% T/I pups. T/I-het mice were phenotypically similar to WT mice and appeared to not be susceptible to developing colitis (Fig 3B). T/I-het females readily became pregnant when co-housed with T/I males and their litter sizes (8 ± 2; Fig 1) were similar to WT mice. T/I and T/I-het littermate pups produced and nursed by T/I-het dams were both normal-sized at weaning (Fig 3A) and did not develop alopecia (0 of 77 litters, 0%).

Overall, the best reproductive performance was obtained using T/I-het dams that were not susceptible to developing colitis. The size of T/I pups at weaning was dependent on dam genotype and health, with the largest pups in litters from T/I-het dams and the smallest in litters from T/I dams. Importantly, use of T/I-het dams that were not colitis-susceptible did not affect the colitis phenotype of their T/I pups. Therefore, based on the higher fertility of T/I-het dams and the enhanced growth and survival of T/I pups when their dams were not colitis-susceptible, we adopted the scheme of breeding T/I males to T/I-het females for routine production and maintaining this line of IBD-susceptible T/I mice.

### Effect of TNF and IL-10 Deficiency on Growth

Weight loss and/or failure to thrive is common in humans when IBD begins in childhood [6,14]. Therefore, we next determined how pup growth was related to pup genotype and colitis susceptibility. T/I and T/I-het littermates born to healthy T/I-het dams were indistinguishable in size and appearance from each other and from WT mice, from birth through weaning at 3 weeks of age (Fig 3A). Mean body weights of T/I-het and T/I mice continued to be similar from weaning until 8 weeks of age, when they diverged and remained significantly different throughout the remainder of the experiment (Fig 3A). Mean body weights of T/I mice became significantly lower than those of WT mice beginning at 13 weeks (Fig 3A). The decreased weight gain observed in T/I mice after 8–9 weeks of age corresponded temporally to the high prevalence of severe UC-like pancolitis at this age, based on data from a separate cohort of mice (Fig 3B).

### Decreased Growth Correlates with Presence of Colon Inflammation

**Fig 4:**
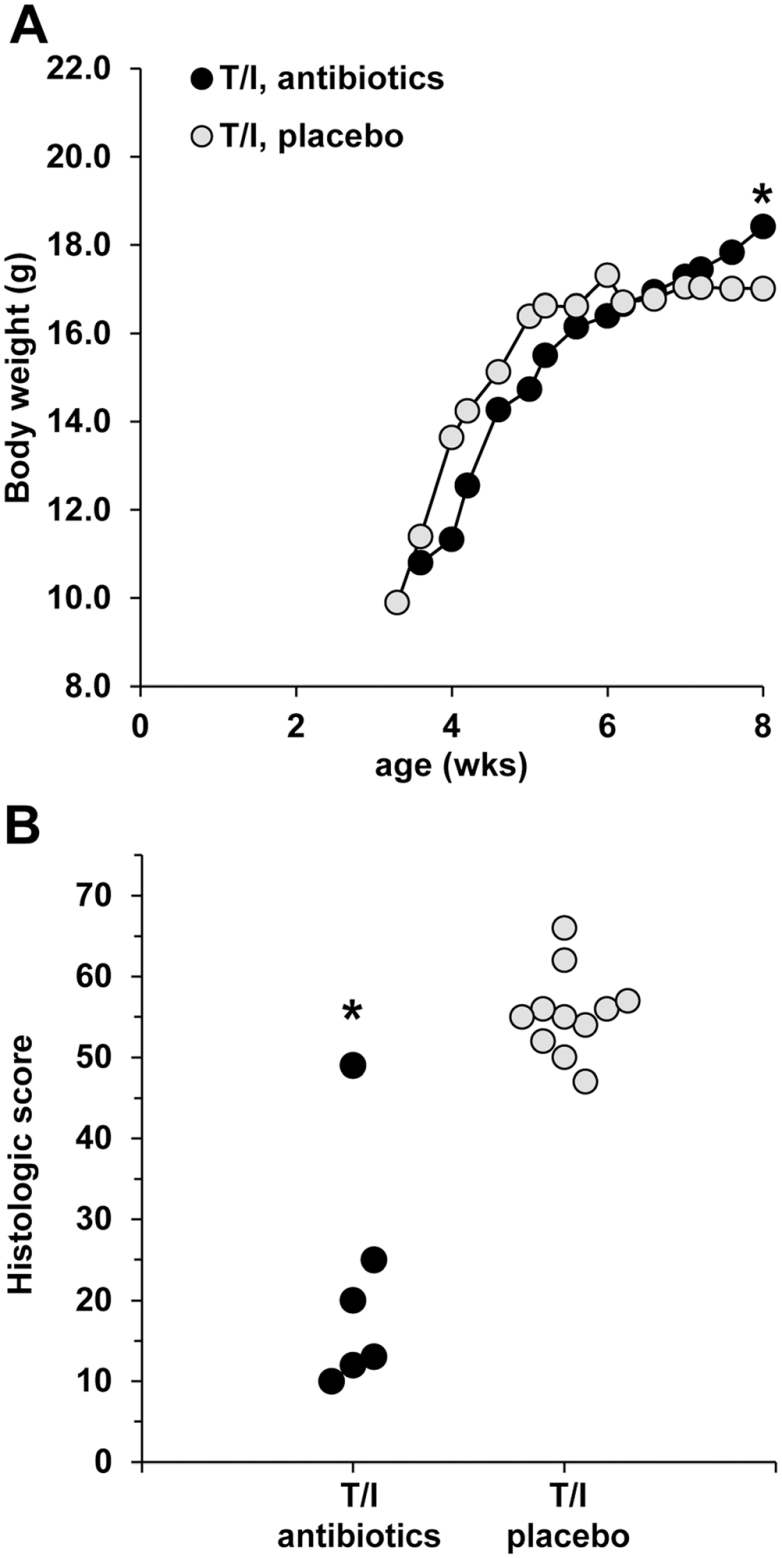
Antibiotic treatment that inhibits the development of colitis prevents growth failure in T/I mice. A. Mean body weight from weaning until 8 weeks of age is shown for female T/I mice that received food containing amoxicillin, clarithromycin, metronidazole, and omeprazole (antibiotics) or matched food lacking these drugs (placebo). The numbers of mice studied were: T/I, antibiotics (n = 6); and T/I, placebo (n = 11). SEMs averaged 0.5 across all days and groups; error bars are omitted for clarity. Although weights for both groups were statistically similar soon after weaning, the weights of the T/I mice that received placebo were significantly lower at 8 weeks of age than those for T/I mice that received antibiotics (p = 0.04; Student’s t-test). B. Colitis histologic scores are shown for these same mice. Each point represents a single mouse studied. A score of ≤12 indicates absence of colitis, while scores ≥ 25 indicate moderate to severe colitis. * indicates a significant difference from mice treated with placebo; p = 0.0003 (Mann-Whitney non-parametric test).

To determine whether the decreased growth in T/I mice was due to the development of colitis, we assessed growth curves of T/I mice in the presence and absence of antibiotic treatment. As shown in Fig 4A, continuous treatment with amoxicillin, clarithromycin, metronidazole, and omeprazole beginning at the time of weaning generated body weights that were significantly higher at 8 wks of age in T/I mice that received the antibiotic cocktail as compared to T/I mice that received placebo (p = 0.04). As we showed previously (9), treatment with the 4 drug cocktail inhibited the development of colitis in T/I mice (p = 0.0003; Fig 4B). Antibiotic treatment had no effect on weight gain in T/I-het mice that were not susceptible to developing colon inflammation (S2 Fig). Thus, the growth failure observed is associated with the development of colitis, which can be modified by antibiotics that affect the microbiota present in the colon.

### Mechanisms for Decreased Growth: Systemic Cytokines

Impaired weight gain in children with UC has previously been attributed to decreased oral intake due to hypothesized changes in appetite (e.g. anorexia or early satiety), nausea mediated by systemic cytokines leading to nutritional deficiency, or to complications of chronic daily corticosteroid administration [6,14]. The pro-inflammatory cytokine TNF has been well-established to play a critical role in the regulatory networks that govern IBD activity in humans [15–17], as well as to decrease appetite and enhance development of cachexia (weight loss and wasting) [18–20]. Due to the genetic absence of TNF in both T/I mice that developed colitis and their T/I-het littermates that did not, use of these mouse strains allowed us to assess factors other than TNF that may affect food consumption and growth.

To assess the potential role of non-TNF pro-inflammatory cytokines, serum cytokines were compared in 9–11 week-old T/I-het and T/I mice (Table 1). As expected, T/I mice had significantly increased serum levels of the Th17 cytokine IL-17, with a strong trend to increased IL-12 p40 (a subunit shared between IL-12 and IL-23), consistent with increased Th17-type inflammation as described previously [9]. Levels of IL-6, IL-13, IFN-γ, and G-CSF were also elevated (Table 1), consistent with active colon inflammation. The chemokines IP-10, KC, MIP-1α, and MIG were elevated, while MCP-1 and MIP-1β were decreased in the serum of T/I mice as compared to T/I-het mice that did not have colitis (Table 2).

**Table 1:** Serum Cytokines in T/I-het vs. T/I Mice at 9 weeks of age**.

	T/I-het (n = 13)	T/I (n = 28)	p-value
IFNγ	4 ± 1	87 ± 22*	0.0008*
IL-1α	380 ± 141	178 ± 64	0.21
IL-5	15 ± 4	3 ± 0*	0.005*
IL-6	5 ± 1	20 ± 3*	8 x 10^−6^*
IL-9	51 ± 5	102 ± 25	0.06
IL-12 (p40)	11 ± 2	19 ± 4	0.07
IL-12 (p70)	22 ± 10	11 ± 5	0.35
IL-13	104 ± 12	191 ± 35*	0.02*
IL-15	103 ± 49	117 ± 51	0.85
IL-17	3 ± 1	39 ± 8*	0.0001*
G-CSF	475 ± 94	5060 ± 618*	9 x 10^−6^*
GM-CSF	10 ± 3	17 ± 2	0.06
M-CSF	15 ± 2	40 ± 18	0.18

* Indicates significant difference (p < 0.05) compared with T/I-het (Student’s t-test).

** Concentrations of each cytokine are presented as mean ± SEM in pg/ml. The following cytokines were present at <10 pg/ml and did not differ between T/I-het and T/I mice: IL-1β, IL-2, IL-3, IL-4, IL-7, VEGF, LIF, IL-10, and TNF.

**Table 2:** Serum Chemokines in T/I-het vs. T/I Mice at 9 weeks of age**.

	T/I-het (n = 13)	T/I (n = 28)	p value
Eotaxin	1040 ± 124	968 ± 56	0.60
IP-10	143 ± 26	337 ± 31*	3 x 10^−5^
KC	128 ± 11	257 ± 42*	0.005
MCP-1	54 ± 9	27 ± 3*	0.01
MIP-1a	19 ± 3	32 ± 3*	0.002
MIP-1b	35 ± 4	15 ± 1*	0.0001
MIP-2	85 ± 7	93 ± 20	0.71
MIG	63 ± 10	474 ± 100*	0.0003
RANTES	38 ± 6	52 ± 11	0.25
LIX	5303 ± 726	5016 ± 311	0.73

* Indicates p < 0.05 compared with T/I-het (Student’s t-test).

** Concentrations of each chemokine are presented as mean ± SEM in pg/ml.

**Fig 5:**
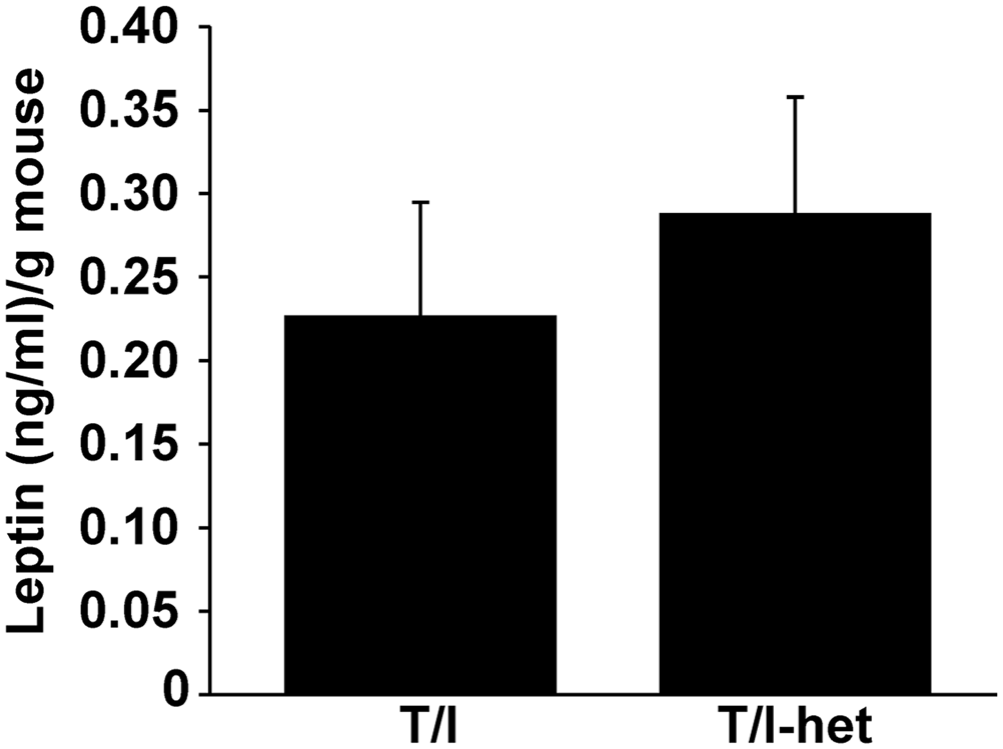
Systemic levels of leptin in T/I-het vs. T/I mice. Mean ± standard deviation of leptin levels did not differ between T/I-het (n = 7) and T/I (n = 11) mice when normalized to body weight (p = 0.08; Student’s t test).

The peptide hormone leptin is secreted from adipocytes in proportion to adipocyte mass and is therefore generally decreased in states of undernutrition. Leptin has been shown to specifically regulate food consumption in addition to its pleotropic effects on metabolism and immune responses [21]. Serum leptin levels were compared in a cohort of T/I mice and their T/I-het littermates, at the time when the growth curves begin to diverge at 9–11 weeks of age. Leptin levels were similar in mice of both genotypes (Fig 5). This was somewhat unexpected, since leptin is both altered by changes in nutritional status and is known to be a pro-inflammatory cytokine [22, 23]. However, these results provide strong support for the hypothesis that the decreased growth observed in T/I mice was due to mechanisms other than decreased food consumption mediated by increased leptin.

### Mechanisms for Decreased Growth: Food Consumption

To rigorously assess possible genotypic or colitis-related effects, food consumed by socially housed WT, T/I-het, and T/I mice was assessed from 3 to 28 wks of age. Results showed that T/I mice actually consumed significantly more food per g body weight than either WT or T/I-het mice (Table 3), despite a significantly lower weight gain over the same period (Fig 3A). Thus, the mechanism for decreased growth in T/I mice with early life onset UC-like colitis is not due to decreased food consumption, as has been previously hypothesized for human children with UC.

**Table 3:** Food Consumption by Mice with Varying Levels of TNF and IL-10 Deficiency.

		Food Consumed	
Genotype	Sex	(mean g/g body weight/day ± SD)	n
WT	F	0.15 ± 0.02 g	62
T/I-het	F	0.13 ± 0.03 g*	63
T/I	F	0.19 ± 0.04 g*	63
WT	M	0.11 ± 0.04 g	60
T/I-het	M	0.13 ± 0.02 g*	63
T/I	M	0.15 ± 0.05 g*	60

* Indicates p ≤ 0.0001 compared with same sex WT mice (ANOVA with Dunnett’s post-test).

### Mechanisms for Decreased Growth: Malabsorption

**Fig 6:**
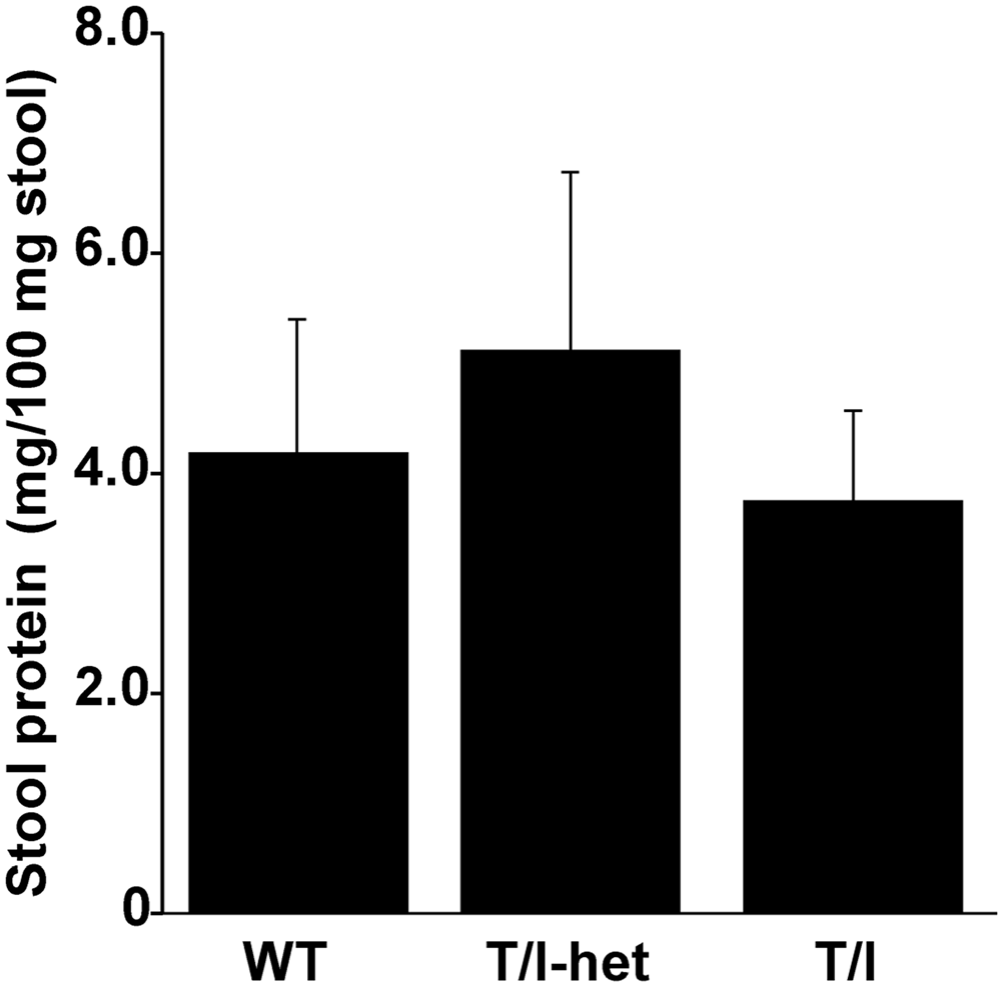
Stool protein in WT, T-het/I, and T/I mice. Mean ± SEM of protein in the stool was similar in WT (n = 12), T/I-het (n = 10), and T/I mice (n = 21) at 9–11 weeks of age (ANOVA with Tukey’s post-test).

Humans with IBD may have low serum protein levels (e.g. albumin) [24] which is often assumed to be due to damage to mucosa that results in a protein-losing enteropathy. To assess this in this mouse model, stool protein content was compared in 9–11 wk old WT, T/I-het, and T/I mice. WT and T/I-het mice did not have colitis at the time of measurement, while T/I mice had moderate to severe colitis. Despite this difference in colon inflammation that affected mucosal integrity, the amount of protein in the stool did not differ between mice of these 3 genotypes (Fig 6). Thus, the mechanism for decreased growth in T/I mice is not due to protein loss in the stool, leading to malnutrition due to protein deficiency.

### T/I mice Show Increased Metabolic Rate *in vivo*

To identify metabolic mechanisms that could account for the growth failure observed in T/I mice, a panel of biochemical and physiologic parameters indicative of metabolic rate were compared in T/I mice and their T/I-het littermates, using a CLAMS metabolic chamber. Once animals had become habituated to the chamber (S1 Fig), the volume of oxygen consumed (VO_2_, ml/kg/hr), the volume of CO_2_ produced (VCO_2_, ml/kg/hr), the respiratory exchange ratio (VCO_2_/VO_2_), and heat production (kcal/kg/hr) were found to not differ between the light and dark cycles for either genotype. Results for each of these parameters were thus collapsed to provide a mean hourly value based on the entire 72 hrs tested (n = 72 observations/mouse). Motor activity, as defined by beam-breaks, and consumption of food and water were higher during the dark cycle, as expected [25, 26], and are presented as daily average values.

**Fig 7:**
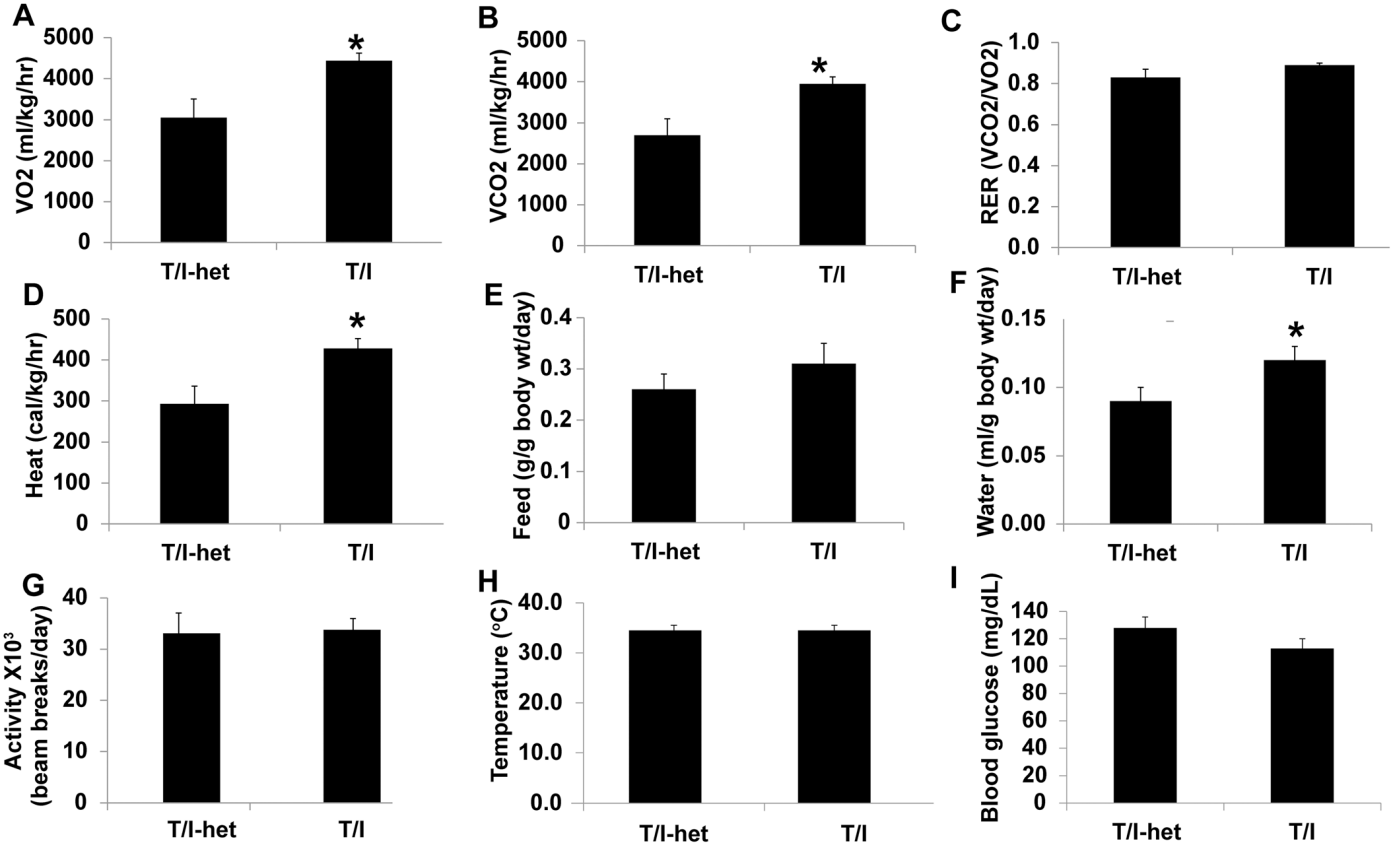
Biochemical and physiologic parameters related to metabolic rate in T/I vs. T/I-het mice. 8–9 wk T/I and T/I-het male mice were acclimated to single housing in a CLAMS metabolic chamber for 4 days, then measurements were recorded continuously over the next 72 hrs. Values shown are mean ± standard error of the mean for 8 mice per genotype. A. Oxygen consumption, VO_2_; B. CO_2_ production, VCO_2_; C. Respiratory exchange ratio (RER); D. Heat production; E. Food consumption; F. Water consumption; G. Motor activity. Additional measurements made outside of the CLAMS unit included body temperatures (H) and blood glucose measurements (I). * indicates p< 0.05.

T/I mice showed increased oxygen consumption (VO_2_, Fig 7A) and production of CO_2_ (VCO_2_, Fig 7B) relative to T/I-het mice. Since both of these parameters were enhanced, the respiratory exchange ratio was similar for both genotypes (Fig 7C), indicating that respiration was higher in T/I than T/I-het mice and this effect was not due to altered substrate utilization of nutrients. Heat production by T/I mice was also significantly increased relative to T/I-het mice (Fig 7D). The T/I mice showed a strong trend toward increased food consumption, but this was not statistically significant over the 3 days analyzed in this study (p = 0.06; Fig 7E). However, water consumption was significantly increased in T/I mice compared to their T/I-het littermates (Fig 7F). Importantly, there was no difference in motor activity between T/I and T/I-het mice (Fig 7G). Body temperature (based on 6–7 separate weekly measurements per mouse for n = 8 mice/group) and blood glucose measurements (n = 8/group, obtained once at the completion of the metabolic chamber studies) were also similar between mice of these 2 genotypes (Fig 7H and 7I).

The increased water consumption by T/I mice was not due to excessive water loss in the stool, as T/I stool had similar water content by weight (52 ± 6%; n = 6) as stool from T/I-het mice (47 ± 3%; n = 6; p = 0.57, Student’s t-test). Urine production was also similar for the two genotypes and averaged 2.1 ± 0.5 ml/mouse/day for T/I mice and 1.8 ± 0.5 ml/mouse/day for T/I-het mice (n = 24 mouse-days for each; p = 0.71; t-test).

### *In vitro* Metabolic Testing

**Fig 8:**
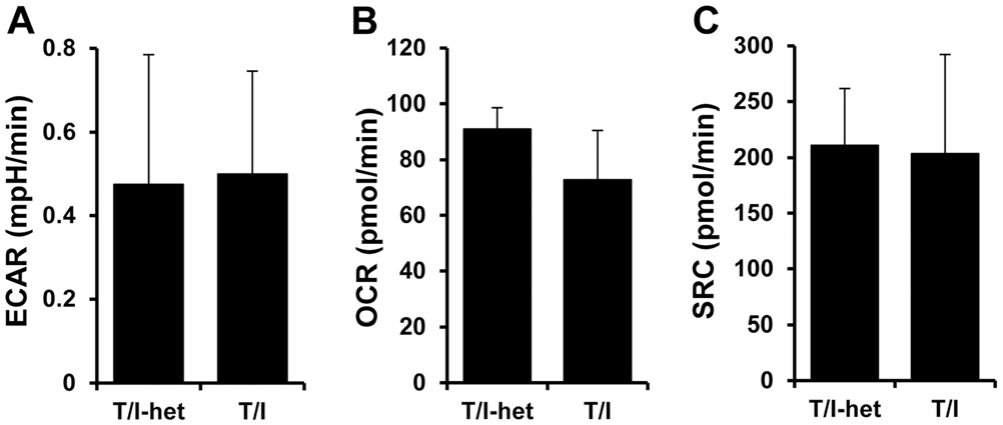
T cell metabolism is not altered in T/I mice with IBD. CD4^+^ T cells were isolated from spleen and mesenteric lymph nodes of T/I-het and T/I mice. Extracellular acidification rate (ECAR, panel A), basal oxygen consumption rate (OCR, panel B), and spare respiratory capacity (SRC, panel C) were measured using a Seahorse Extracellular Flux Analyzer.

Due to ongoing inflammation, T/I mice have activated immune cells present within their colonic mucosa as well as in other lymphoid organs such as mesenteric lymph node and spleen. Activated effector T cells are required for IBD pathogenesis and effector T cell activation is accompanied by increased cellular metabolism, characterized by a predominant shift in T cell glucose uptake and glucose metabolism [27]. To assess whether dual deficiency of TNF and IL-10 or ongoing severe colitis affected the metabolism of these immune cells, the oxygen consumption rate (OCR, a measure of mitochondrial oxidation processes) and the extracellular acidification rate (ECAR, an indicator of glycolysis) were measured in CD4^+^ T lymphocytes derived from the spleen and the mesenteric lymph node. Based on the OCR (Fig 8A) and ECAR (Fig 8B), no differences in energy usage on a per cell basis were seen in lymphocytes obtained from T/I mice and their T/I-het littermates. In addition, there were no differences in spare respiratory capacity in T/I mice compared to T/I-het littermates (Fig 8C). Thus, a cell-intrinsic alteration in lymphocyte metabolism does not appear to account for the changes in metabolic rate seen in T/I mice with active disease.

## Discussion

In this murine model of IBD, we found that T/I-het dams with a genotype that made them unlikely to develop colitis demonstrated better reproductive performance. Colitis-susceptible T/I pups born to healthy (T/I-het) dams initially gained weight normally, however their growth curves had diverged from their colitis-resistant T/I-het littermates by 8 weeks, an age by which we have previously shown the near universal development of IBD in mice of this genotype [9]. Administration of an antibiotic cocktail that prevented development of colitis also normalized the T/I growth curve. Failure of weight gain occurred in T/I mice that developed colitis despite a significant increase in their food consumption. It was thus not due to TNF-induced anorexia since TNF was genetically absent or to elevated leptin levels as has been hypothesized for human children with UC, since T/I mice ate more chow than their T/I-het littermates or WT mice. The protein content of stool was similar in T/I, T/I-het, and WT mice, further excluding protein-losing enteropathy as a mechanism for failure of T/I mice to gain weight. Taken together, these data pointed to more complex mechanisms for growth failure in T/I mice with early life onset of IBD.

A few human studies have assessed differences in energy expenditure in patients with IBD compared to controls. Patients with CD have generally been found to have an increase in energy expenditure [28–30]. Treatment with immunosuppression or with surgical resection partially reverses or normalizes this increased energy expenditure [28–29]. Mechanisms for altered energy expenditure following treatment remain unknown and may be via direct action on inflammation or through indirect effects on body composition characteristics. Our metabolic chamber studies demonstrated that T/I mice had increased oxygen and water consumption and increased CO_2_ and heat production, while body temperatures, motor activity, and blood glucose levels were similar to those of the control T/I-het mice. Based on their phenotypic similarities to human IBD patients, T/I mice may provide a useful model to better determine mechanisms of altered energy expenditure in IBD.

Although most T/I mice had demonstrated an IBD-associated decline in their trajectory of weight gain when they reached reproductive age, they typically did not demonstrate other obvious behavioral or physical signs of illness. Nevertheless, T/I dams generally exhibited poor reproductive performance. Active IBD in humans has been similarly linked to adverse pregnancy outcomes. Women with active IBD experience lower rates of conception and higher risk of pregnancy- and delivery-related complications, including spontaneous abortions, preterm deliveries, prematurity, low birth weight and caesarean section [31–33]. Disease activity at conception has been associated with fetal loss, with flares later in gestation also associated with preterm birth and low birth weight [32]. The work reported here sought to maximize production of T/I pups through optimization of breeding strategies. However, based on its similarity with what has been observed in humans, studies using T/I dams appear to be highly suited for investigation of IBD-related mechanisms that may affect fertility, including reproductive cycling, mating behavior, spontaneous abortions, and lactation failure. Studies using T/I dams may also be useful for testing novel interventions that could improve reproductive success in the setting of IBD.

Their non-circadian increases in oxygen and water consumption and in heat and CO_2_ production in the absence of changes in body temperature or motor activity suggest that T/I mice have an increased metabolic rate. The relatively small differences in daily feed consumption observed in male T/I vs. T/I-het mice in the long term studies (Table 3) likely account for the lack of significant differences in food consumption measured during the 3 days that male T/I and T/I-het mice were studied in the CLAMS metabolic chamber. That T/I mice drank significantly more water was unexpected. Their urine production was similar to that of T/I-het mice, while random non-fasting glucose measurements were normal, ruling out a polydipsia/polyuria sequence due to glycosuria (e.g. diabetes mellitus). Increased water consumption was not required to compensate for stool-related losses of water, since stool water content was similar for T/I and T/I-het mice. CD4+ lymphocytes isolated from T/I or T/I-het control mice failed to show significant differences in oxidative or glycolytic activity on a per cell basis, suggesting an absence of cell-intrinsic metabolic differences in the two strains. Measurement of changes in metabolism of innate immune cells, brown or white adipose tissue, or muscle were beyond the scope of the current study. However, the greater water intake of T/I mice may have simply reflected a greater need for water due to their increased heat production [34]. Additional studies will be needed to determine the precise mechanisms that led to the increased metabolic rate observed in T/I mice.

Approximately one-third of human patients with IBD first become symptomatic in childhood or adolescence [35]. Since weight loss is observed before diagnosis in 60% of children with UC [36], these children may present with malnutrition or growth retardation rather than more specific colon-related symptoms and they may also experience delayed pubertal development [6]. Disordered somatic development in children with IBD has been attributed to inflammation, malnutrition caused by increased intestinal losses of protein, and decreased appetite. Affected children may be iron-deficient, since iron deficiency is often associated with loss of blood from the gastrointestinal tract [36]. Others have suggested that growth impairment in UC, if it occurs, is most often a complication of chronic daily corticosteroid administration [14]. Using computed open-circuit indirect calorimetry, Sasaki *et al*. [37] showed that adult patients with moderate to severe UC exhibited a hypermetabolic status compared with healthy controls, with an increased resting energy expenditure normalized to body weight that was significantly correlated with disease activity. However, these patients were older and results may potentially have been confounded by differences in energy sources since patients received total parenteral nutrition, while healthy controls were fasted and since patients but not controls received corticosteroid therapy (30–80 mg/day). Our data show that young mice with untreated early life-onset IBD exhibit growth failure in the absence of corticosteroid administration, without increased protein loss in the stool, and despite increased food consumption.

Although we can exclude an effect of TNF itself on growth failure in the (TNF-deficient) T/I model, the other cytokines and chemokines whose levels are altered in T/I mice have generally not been formally studied for their effects on food consumption, growth, and weight loss. Nevertheless, the current data collectively raise the possibility that inflammation itself, mediated through production of specific inflammatory mediators, the collective metabolism of large numbers of activated immune cells, or other mechanisms, may affect metabolic rate and energy expenditure at levels sufficient to result in IBD-associated growth failure. Pro-inflammatory cytokines have previously been shown to regulate energy metabolism in both physiological and pathological conditions, including obesity, aging (calorie restriction), and response to exercise [38]. Further studies will be needed to elucidate how inflammation may activate or repress specific metabolic pathways and to identify novel or existing therapeutics that can reverse those changes. Analyses of these mechanisms may also aid the development of agents that can treat or prevent obesity. Based on its clinical similarities to human IBD, further studies using the T/I model of colitis may provide unique insights into growth failure and/or delayed puberty that can be translated into improved clinical treatments for these socially distressing complications of IBD in human children.

IBD is currently hypothesized to result from an inappropriate immune response against intestinal microbiota that occurs in a genetically susceptible host. Many studies have documented changes in intestinal microbiota between healthy mice and humans and those with IBD (reviewed in [39]). Although certain microbes have been identified to enhance colon inflammation in susceptible individuals [40–42], the current understanding of whether colitis-associated changes documented in gut microbial communities represent cause or effect of inflammation is still limited. The microbes present in the gut have also been shown to play major previously unrecognized and apparently causative roles in metabolism and energy expenditure [43], including the extreme weight loss seem in kwashiorkor [44] and weight gain in obesity [45]. Whether IBD-associated changes in the gut microbiota may potentially be directly or indirectly responsible for the growth failure we observed in T/I mice is of great interest, but will require additional study. The T/I model provides considerable advantages to address this question, since the near universal spontaneous development of colitis will allow changes in gut microbial communities to be monitored longitudinally before and after the development of colitis and growth failure.

In summary, this study shows that the mechanisms for growth failure in T/I mice with early life onset of UC-like IBD are more complex than previously hypothesized for humans. Due to its strong clinical similarities with human IBD, the T/I murine model can be extremely valuable for further investigation of mechanisms that lead to growth and/or reproductive failure in the setting of IBD. Such investigations can potentially facilitate the development of treatments to improve the quality of life for women and children with IBD.

## Supporting Information

S1 Fig Biochemical and physiologic parameters related to metabolic rate in T/I vs. T/I-het mice, during the acclimation period. 8–9 wk T/I and T/I-het male mice were placed in single housing in a CLAMS metabolic chamber, then measurements were recorded continuously during the 4 day acclimation period. Values shown are mean ± SEM for 8 mice per genotype. A. Oxygen consumption, VO_2_; B. CO_2_ production, VCO_2_; C. Respiratory exchange ratio (RER); D. Heat production; E. Food consumption; F. Water consumption; G. Motor activity. The specific day during the acclimation period is indicated in circles below the horizontal axis, with measurements made during the light cycle indicated by a white circle and measurements made during the dark cycle indicated by a black circle.
(TIF)

S2 Fig Antibiotic treatment that prevents colitis and growth failure in T/I mice has no effect on their T/I-het littermates. A. Mean body weight from weaning until 8 weeks of age was similar for female T/I-het mice that received food containing amoxicillin, clarithromycin, metronidazole, and omeprazole (antibiotics, n = 4) or matched food lacking these drugs (placebo, n = 4). SEMs averaged 0.5 across all days and groups; error bars are omitted for clarity. B. Colitis histologic scores are shown for these same mice. Each point represents a single mouse studied. All mice had a histologic score of ≤12, which indicates absence of colitis.
(TIF)

S1 Table Pathogen status of mice used for these studies. (DOCX)
