# Supplementary figures and images for: Reproduction and Growth in a Murine Model of Early Life-Onset Inflammatory Bowel Disease
Source: PLoS One. 2016 Apr 5;11(4):e0152764. doi: 10.1371/journal.pone.0152764 (PMC4821577; doi:10.1371/journal.pone.0152764)

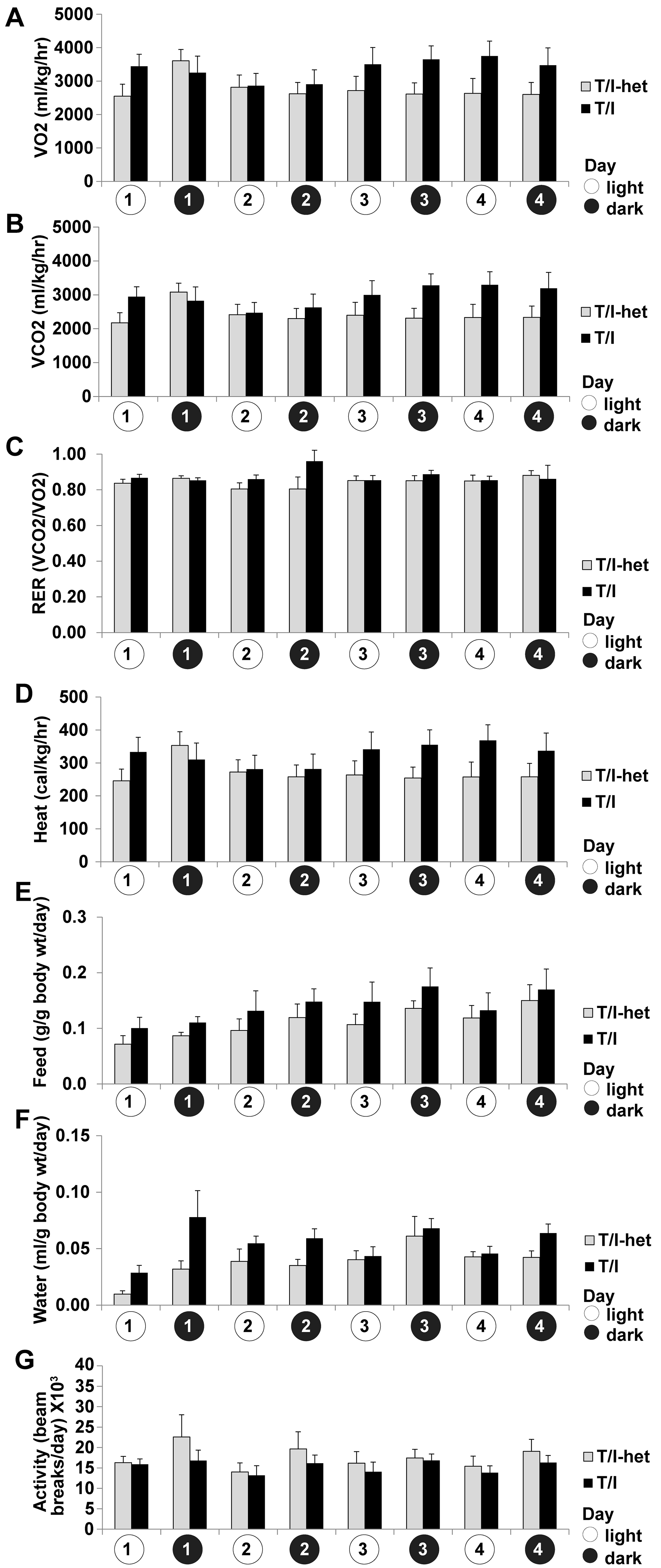

Supplement: S1 Fig — 8–9 wk T/I and T/I-het male mice were placed in single housing in a CLAMS metabolic chamber, then measurements were recorded continuously during the 4 day acclimation period. Values shown are mean ± SEM for 8 mice per genotype. A. Oxygen consumption, VO2; B. CO2 production, VCO2; C. Respiratory exchange ratio (RER); D. Heat production; E. Food consumption; F. Water consumption; G. Motor activity. The specific day during the acclimation period is indicated in circles below the horizontal axis, with measurements made during the light cycle indicated by a white circle and measurements made during the dark cycle indicated by a black circle. (TIF) [file pone.0152764.s001.tif]

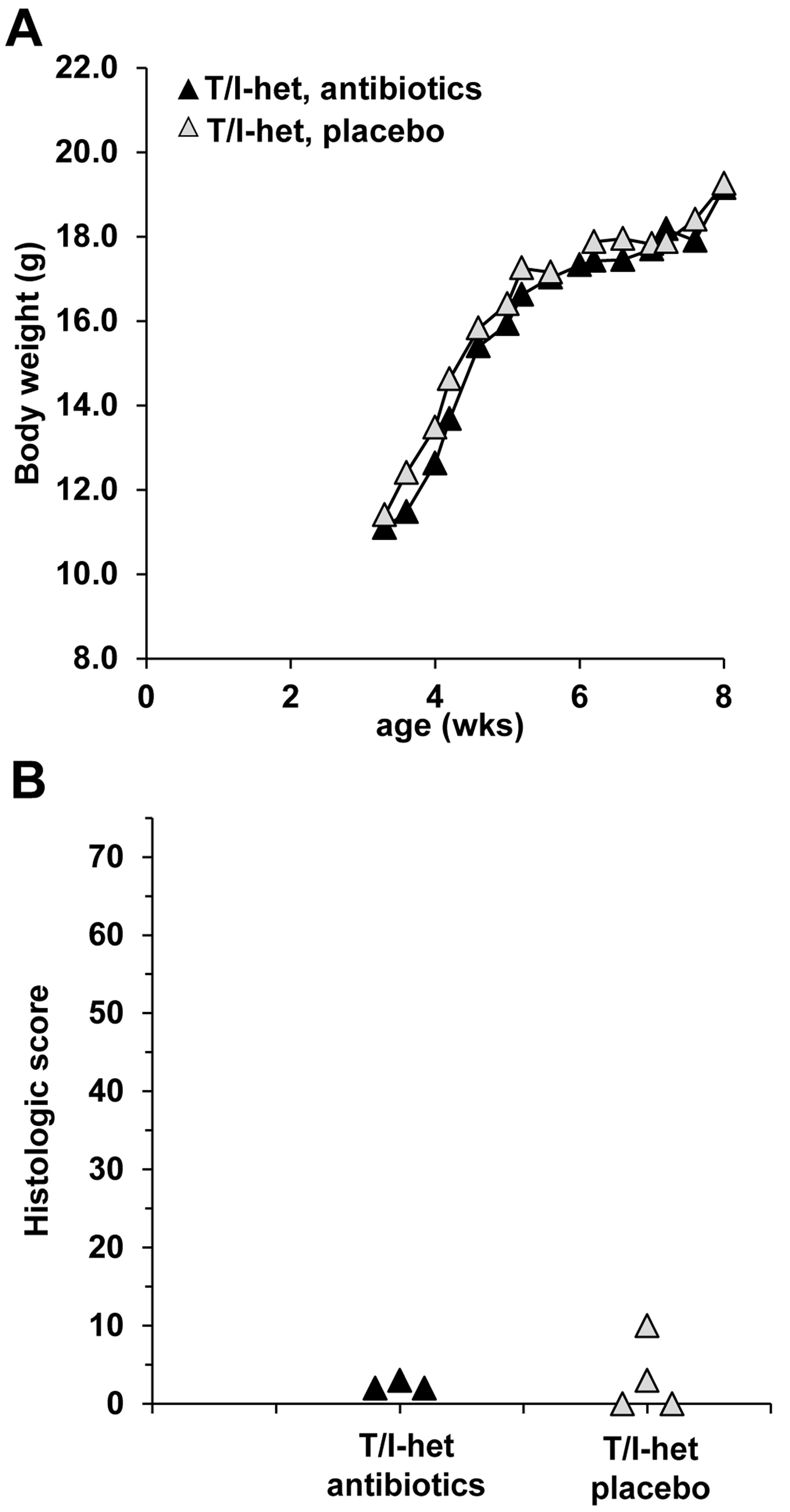

Supplement: S2 Fig — A. Mean body weight from weaning until 8 weeks of age was similar for female T/I-het mice that received food containing amoxicillin, clarithromycin, metronidazole, and omeprazole (antibiotics, n = 4) or matched food lacking these drugs (placebo, n = 4). SEMs averaged 0.5 across all days and groups; error bars are omitted for clarity. B. Colitis histologic scores are shown for these same mice. Each point represents a single mouse studied. All mice had a histologic score of ≤12, which indicates absence of colitis. (TIF) [file pone.0152764.s002.tif]
