# Supplementary material for: Reproduction and Growth in a Murine Model of Early Life-Onset Inflammatory Bowel Disease
Source: PLoS One. 2016 Apr 5;11(4):e0152764. doi: 10.1371/journal.pone.0152764 (PMC4821577; doi:10.1371/journal.pone.0152764)
Supplement: S1 Table — (DOCX) [file pone.0152764.s003.docx]

**S1 Table**

**Pathogen Status of Mice Used for These Studies**

| **Pathogen** | **Status** | **Test Type** |
| --- | --- | --- |
| *Syphacia obvelata* | Negative | Tape test, fecal float |
| *Aspicularis tetraptera* | Negative | Tape test, fecal float |
| *Myobia musculi* | Negative | Tape test, skin scrape |
| *Mycoptes musculinus* | Negative | Tape test, skin scrape |
| *Radfordia affinis* | Negative | Tape test, skin scrape |
| Nits | Negative | Skin scrape |
| Other ectoparasites | Negative | Skin scrape |
| Other endoparasites | Negative | Fecal float |
| Mouse thymic virus (MTV) | Negative | Serology |
| Cilia-associated respiratory bacillus | Negative | Serology |
| *Encephalitozoon cuniculi* | Negative | Serology |
| Ectromelia | Negative | Serology |
| Epizootic diarrhea of infant mice virus | Negative | Serology |
| Hantaan virus | Negative | Serology |
| Mouse pneumonitis virus (K virus) | Negative | Serology |
| Lymphocytic choriomeningitis virus | Negative | Serology |
| *Mycoplasma pulmonis* | Negative | Serology |
| Mouse adenovirus strain 1 (FL) | Negative | Serology |
| Mouse adenovirus strain 2 (K87) | Negative | Serology |
| Mouse cytomegalovirus (MCMV) | Negative | Serology |
| Mouse hepatitis virus (MHV) | Negative | Serology |
| Murine norovirus (MNV) | Negative | Serology |
| Minute virus of mice (MVM) | Negative | Serology |
| Mouse parvovirus (MPV) | Negative | Serology |
| Polyoma virus | Negative | Serology |
| Pneumonia virus of mice (PVM) | Negative | Serology |
| Reovirus type 3 | Negative | Serology |
| Theiler's murine encephalomyelitis virus (TMEV)- strain GDVII | Negative | Serology |
| Sendai virus | Negative | Serology |
| Helicobacter species | Negative | Fecal PCR |
| *Pasteurella pneumotropica* | Negative | Fecal PCR |
| *Citrobacter rodentium* | Negative | Fecal culture |
